# The Importance of Landscape Elements for Bat Activity and Species Richness in Agricultural Areas

**DOI:** 10.1371/journal.pone.0134443

**Published:** 2015-07-31

**Authors:** Olga Heim, Julia T. Treitler, Marco Tschapka, Mirjam Knörnschild, Kirsten Jung

**Affiliations:** 1 Institute of Evolutionary Ecology and Conservation Genomics, University of Ulm, Ulm, Germany; 2 Smithsonian Tropical Research Institute, Balboa, Panama; Università degli Studi di Napoli Federico II, ITALY

## Abstract

Landscape heterogeneity is regarded as a key factor for maintaining biodiversity and ecosystem function in production landscapes. We investigated whether grassland sites at close vicinity to forested areas are more frequently used by bats. Considering that bats are important consumers of herbivorous insects, including agricultural pest, this is important for sustainable land management. Bat activity and species richness were assessed using repeated monitoring from May to September in 2010 with acoustic monitoring surveys on 50 grassland sites in the Biosphere Reserve Schorfheide-Chorin (North-East Germany). Using spatial analysis (GIS), we measured the closest distance of each grassland site to potentially connecting landscape elements (e.g., trees, linear vegetation, groves, running and standing water). In addition, we assessed the distance to and the percent land cover of forest remnants and urban areas in a 200 m buffer around the recording sites to address differences in the local landscape setting. Species richness and bat activity increased significantly with higher forest land cover in the 200 m buffer and at smaller distance to forested areas. Moreover, species richness increased in proximity to tree groves. Larger amount of forest land cover and smaller distance to forest also resulted in a higher activity of bats on grassland sites in the beginning of the year during May, June and July. Landscape elements near grassland sites also influenced species composition of bats and species richness of functional groups (open, edge and narrow space foragers). Our results highlight the importance of forested areas, and suggest that agricultural grasslands that are closer to forest remnants might be better buffered against outbreaks of agricultural pest insects due to higher species richness and higher bat activity. Furthermore, our data reveals that even for highly mobile species such as bats, a very dense network of connecting elements within the landscape is beneficial to promote activity in open areas and thus assure vital ecosystem function in agricultural landscapes.

## Introduction

Within the cultural landscapes of Central Europe, natural vegetation cover has been largely replaced by managed agricultural areas [1] and fragments of production forest systems [2–3]. The intensification of land use and the enlargement of agricultural fields and cattle pastures in the last century have caused drastic changes in the European landscape matrix [4–6]. Formally heterogeneous and complex landscape mosaics have been transformed into homogenous agricultural regions containing little or no refuge zones for biodiversity [4, 7]. As a consequence, many species of vascular plants [8], invertebrates [9–10] and vertebrates [11–13] experienced drastic population declines in the last decades, with detrimental effects on their contributing ecosystem services [7, 14] such as pollination or pest control [15].

Key structures for maintaining or enhancing structural complexity and thus local biodiversity and ecosystem service function in agro-ecosystems are landscape elements [7] such as single trees [16], hedges [17] or water bodies [18], as they provide food [19], shelter, breeding and roosting opportunities [20] for many animal species. In addition, such landscape elements can serve as corridors and stepping stones connecting suitable habitat patches for many wildlife species [21] with remnants of mature forested areas which might provide a source for species diversity due to higher habitat heterogeneity [22–23]. A well-connected network of such landscape elements thus seems essential for species to capitalize on potential resources [24] within homogenously farmed agricultural landscapes. Hence, it is important to understand the effect of different connecting landscape elements on species distribution, and their potential to buffer species declines and decreasing ecosystem service contributions in agricultural landscapes.

Landscape connectivity is considered to enhance the movement of species and individuals across space and varies strongly as a function of species mobility [25] and the ability of organisms to use certain landscape features. European bats are generally very mobile and provide important ecosystem services by controlling many herbivorous insects in forests [26] and agricultural systems [27–28]. Their persistence in human dominated landscapes therefore is of direct interest to sustainable landscape management and insect pest control in agricultural areas [29]. However, foraging away from vegetation cover might impose a higher predation risk and higher energetic costs of flight to bats due to a lack of cover from predators or stronger wind, especially for slower flying bat species. In addition, higher frequency echolocation limits the perceptual range of prey detection and thus foraging of bats using higher echolocation calls may be less effective in wide open areas. Therefore, both high flight speeds [30] (which reduce the risk of predation and favor a quick pursuit of insects after prey detection) and lower echolocation frequencies (which are less affected by atmospheric attenuation) are beneficial for bats in open areas.

Throughout evolution, bat species have adapted their flight and echolocation performance to sensorial constraints imposed by the acoustical clutter of their foraging habitats, and can be classified into functional groups according to their ability to forage in open, edge and narrow spaces (e.g. [31]). Previous studies have indeed shown that activity of functional groups in open areas differs in relation to the presence of landscape elements [32]. Single trees [33–35], tree lines, hedges and forest remnants within the landscape are known to enhance overall bat activity [32, 34] and species richness [22]. However, it remains unclear whether the importance of landscape elements in the direct vicinity of agricultural areas might vary throughout the year, and thus whether potential ecosystem service contributions vary in time.

Here we investigated how species richness, bat activity, and species composition above grasslands are influenced by the local landscape setting. We hypothesized that bat activity and species richness above grassland areas would increase with proximity to forest and anthropogenic areas, because both potentially provide roosting opportunities and enhance heterogeneity. In addition, we expected a positive effect of landscape elements such as single trees or tree lines for bat activity on grassland sites because these may connect open landscapes with forested or urban areas. Specifically, we expected that the importance of forested areas should be more important under less favorable climatic conditions in spring and autumn. Finally, we hypothesized that the landscape elements, due to species-specific challenges imposed by their flight and echolocation performance, influence species composition. Specifically we expected that the activity of open space foraging bats should be rather independent of forested areas and connecting landscape elements, in contrast to edge and narrow space foraging species.

## Materials and Methods

### Ethics statement

Permission for field work was granted by the Landesumweltamt Brandenburg, Germany (permit number: RO7/SOB-02). Free ranging bats where surveyed using passive acoustic monitoring and thus not affected in any way.

### Study area

**Fig 1:**
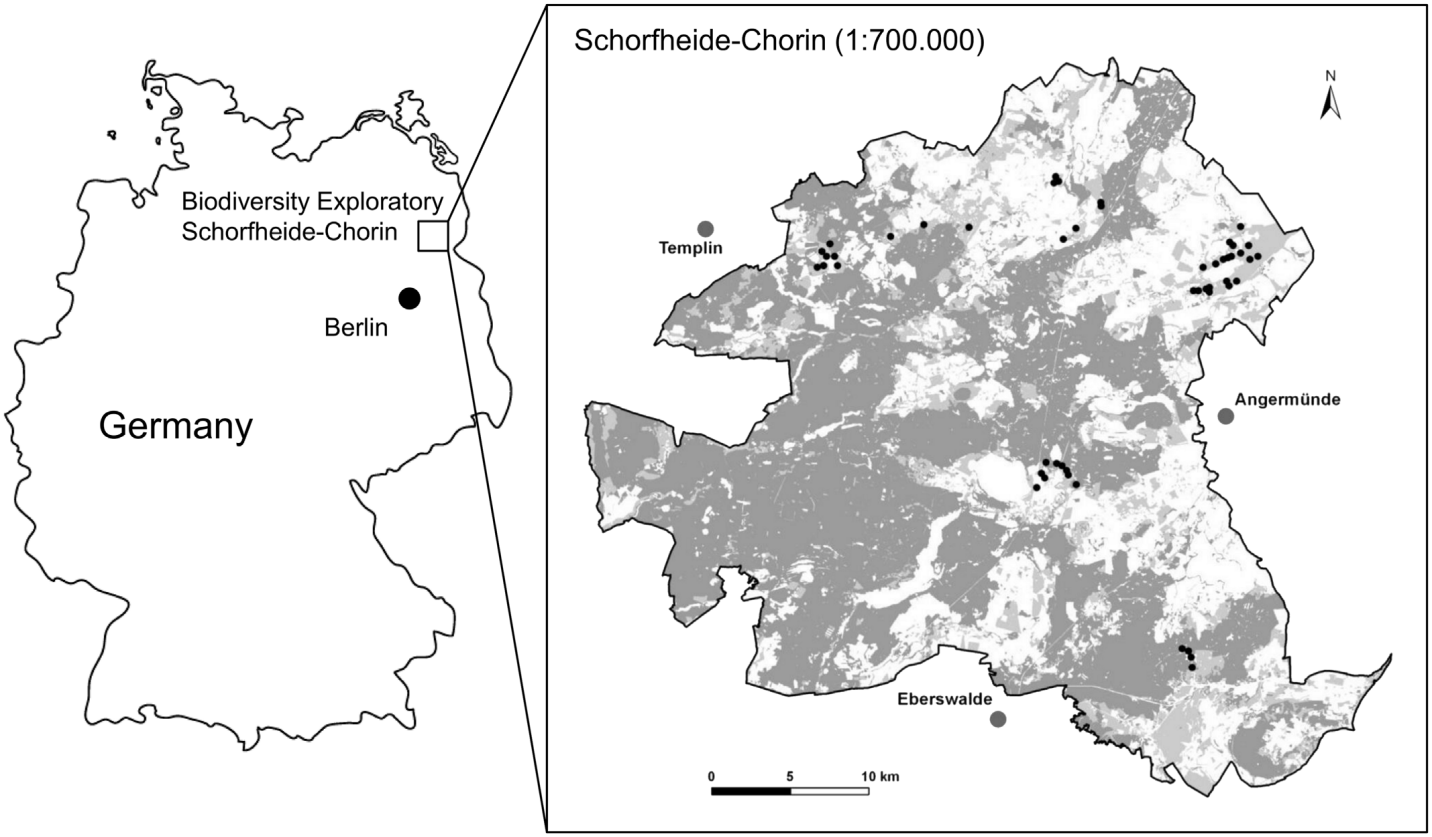
Location of the Biodiversity Exploratory Schorfheide-Chorin in Germany and a map of the Biosphere Reserve Schorfheide-Chorin. Dark grey areas indicate forest patches, light grey areas and white areas indicate agricultural fields or grassland areas. Black circles indicate the location of the 50 permanent recording sites in grassland where acoustical monitoring of bats took place.

Our study was located within the biosphere reserve Schorfheide-Chorin (1300 km^2^) in the state of Brandenburg, North-East Germany. The Schorfheide-Chorin is a young glacial landscape characterized by moraines, lakes and marshes [36], and low human population density (23/km^2^). Mean annual temperature ranges between 8.0 and 8.5°Celsius and annual precipitation ranges between 520 mm and 600 mm. Large-scale agricultural cultivation, typical of the former German Democratic Republic (GDR), shaped the present-day structure of the landscape matrix of this area [37–38]. In this study, we focused on 50 grassland sites (Fig 1), mainly used as pastures or meadows for hay production, which differed in their distance towards forested and urban areas, both of which potentially provide roosting opportunities for bats (maximum distance of 500 m to forested and 1.5 km to anthropogenic areas). Grassland sites are part of the large-scale and long-term project “Biodiversity- Exploratories” (www.biodiversity-exploratories.de) on functional biodiversity research in Germany (for details please refer to [37]). Grassland sites have thus been selected in 2006 to obtain experimental sites for long term and comparative biodiversity studies in Germany. Clustering of sites was reduced as much as possible, but was also influenced by the willingness of local stakeholders and land owners to provide access to their land. Spatial clustering of data can pose limitations for regression modeling. We thus tested our data prior to analysis for spatial autocorrelation (please see below).

### Acoustic monitoring of bats

We used stationary automatic ultrasound recording systems (Batcorder, EcoObs GmbH, Marckmann, Schuster and Runkel, Nürnberg, Germany) to record bat occurrence and activity above 50 grassland sites. A Batcorder was installed on top of a 1.80 m pole at the centre of each grassland plot, directing the microphone towards open landscapes. Each grassland plot was sampled five times (one night per month) between May and September 2010, except for 10 plots which could only be accessed four times due to logistic reasons (e.g., grazing bulls, hunting). We surveyed five to six grassland plots simultaneously within the same night and sampling plot combinations were randomized across the season. The recording system was operating from sunset until 01:00 AM to limit recording time to the first peak of bat activity during the night [39–40] and to allow the collection of data and replacement of batcorders on other, and often quite distant, grassland plots for the next night. To control for the potential confounding effect of moonlight, we visited grassland sites only in a two week period just before and after new moon. Recording was aborted in case of rainfall and the survey was repeated the following night.

Recordings were made in real time (sampling rate of 500 kHz, 16 bit) and triggered by the sound pressure level at a threshold of -27 dB SPL. A frequency filter at 15 kHz was used to avoid that environmental noise triggered additional recordings. A recording continued as long as the sound pressure level remained above the threshold level within a post trigger time of 800 ms to assure that complete passes of bats with very long pulse intervals (e.g. *Nyctalus noctula*) would be stored within one file.

### Acoustic data analysis

We assessed the number of bat passes per grassland plot and defined a pass as a sequence of at least two consecutive echolocation calls exceeding the threshold level of -27 dB SPL. Successive passes were discriminated if the time interval between calls exceeded the post trigger time of 800 ms. Mean activity per hour, rounded up to the nearest integer number was then taken as a measure of bat activity per recording night and site. Bat activity and the number of feeding buzzes were significantly positively correlated (Pearson *r* = 0.80, *p* <0.001), indicating that recording sites with higher bat activity were also better foraging habitats for bats. We thus considered bat activity as a measure for the intensity of habitat use on grassland sites. We also considered presence only data of species to assess species richness on grassland plots per sampling event.

### Species identification

Species identifications were assessed by pre-classifying echolocation calls using the automated identification software bcIdent (EcoObs GmbH, Marckmann, Schuster and Runkel, Nürnberg, Germany). In addition, we manually verified all identifications with the software Avisoft SASLab Pro Version 5.1.13 (Avisoft Bioacoustics, Raimund Specht, Berlin Germany) by comparing call structure and frequency to published data in the literature (e.g., [41–47]). Sonograms were generated using a Hamming window, a FFT of 512 points and an overlap of 93.75% (time / frequency resolution: 0.06 ms / 977 Hz). We unambiguously identified echolocation sequences of *Pipistrellus nathusii*, *Pipistrellus pipistrellus*, *Pipistrellus pygmeaus*, *Nyctalus noctula*, *Eptesicus nilssonii*, *Myotis myotis*, *M*. *nattereri* and *Barbastella barbastellus* (S1 Fig). Echolocation calls of *N*. *leisleri* could only be assigned to species level if the typical echolocation sequence of regularly alternating frequencies between 21 kHz to 24 kHz occurred [48]. Furthermore, we did not discriminate between *Plecotus auritus* and *P*. *austriacus* and grouped them to *Plecotus sp*. Finally we assigned echolocation sequences with very similar echolocation calls structure and frequency (14.6% of our data) to the sonotypes *Nyctaloid* low, *Nyctaloid* high and *Myotis sp*. (for more information regarding species identification please refer to S1 Fig). Following Schnitzler & Kalko [31], we classified bats according to their predominant foraging space into narrow space foragers (genera: *Myotis* and *Plecotus*), edge space foragers (genus: *Pipistrellus*) and open space foragers (genera: *Barbastella*, *Eptesicus*, *Nyctalus* and the sonotype *Nyctaloid*).

### Assessment of the landscape matrix

Based on a digital landscape model (Version 2009, resolution: 1:10000, Landesvermessungsamt Brandenburg) and aerial photographs of the region (taken in 2009), we assessed the closest distance from each grassland recording site to landscape elements such as single trees, linear vegetation (tree- / hedgerows), groves (tree groups), running water elements (streams / rills), and water surfaces (lakes / ponds) using ArcGIS 9.31 (ESRI, Redlands, California). All of these landscape elements represent potential connecting elements in open landscapes. In addition, we assessed the distance to and the percent land cover of forests and urban areas within a 200 m buffer around the grassland site to represent differences in the local landscape setting.

### Statistical data analysis

To assess which landscape elements are most important for species richness and higher bat activity on grassland recording sites, we performed generalized linear mixed effect models (glmer, R-package lme4, [49]). For this analysis, we used the accumulated presence only data of species occurring per recording site (species richness) and month, and general activity per recording site and month as dependent variables. Distance to landscape elements and the percent land cover of urban areas and forest in the 200 m buffer were included as explanatory variables into the model. Recording sites were included as random effect, because we repeatedly visited each recording site from May to September.

We further assessed whether forest area in the 200 m buffer might be especially important for species richness and bat activity in different months throughout the time of the year that bats are active. To this end, we divided recording sites based on the mean forest area within the 200 m buffer (mean = 12%) into two subsets with 1.) a higher amount of forest (range: 13–68%, N = 17) and 2.) a lower amount of forest (range: 0–12%, N = 33). A generalized linear mixed effect model (glmer, R-package, lme4) including recording sites as random factor, with forest amount categories interacting with the recording month was then used to assess a seasonal variation in the importance of forest areas.

For all generalized linear mixed effect models, we used a Poisson distribution due to count data. In addition, prior to all analysis we used Moran’s I to reject the possibility for spatial autocorrelation of variance in bat activity (Moran’s I: 0.003; p> 0.79) and species richness (Moran’s I: 1.53*10^−6^, p> 0.87) between recording sites.

To investigate differences in species composition weighted by activity (based on Bray-Curtis dissimilarity) between grassland sites, we performed a non-metric multidimensional scaling (NMDS, with 1000 permutations, R-package vegan, [50]). To evaluate how landscape elements correlated with such differences in bat species activity, we used environmental fitting (envfit, R-package vegan, p-values based on 1000 permutations). Finally, we used a permutational multivariate analysis of variance based on distance matrices and 1000 permutations (R-package vegan, function adonis) to assess whether species richness within functional groups might be determined by different landscape elements.

Neither bat species composition weighted by activity (Pearson *r* = 0.08, *p* > 0.05) nor species composition weighted by species occurrence (Pearson *r* = 0.04, *p* > 0.05) correlated with geographical proximity of sampling sites (Mantel tests, based on a Pearson product moment correlation of dissimilarity matrices and 1000 permutations; R-package vegan).

## Results

### Species richness and bat activity

In total, we obtained 18,055 bat passes over the grassland sites of the Schorfheide-Chorin during five months of data collection from May to September. The highest activity of bats was registered on a grassland plot with the smallest distance to forest (41 m) and the greatest forest land cover (68%) within the 200 m buffer zone. In contrast, activity and species richness were lowest at one of the most isolated grassland plots, in an open landscape at a distance of about 250 m to a linear vegetation element and 500 m to the closest forest patch.

### Importance of landscape elements for bat activity and species richness

As expected, landscape elements revealed a high importance for increased species richness and bat activity above grassland sites (Table 1). In particular, greater forest land cover (*p* < 0.01) and smaller distance (*p* < 0.05) to forested areas significantly promoted species richness of bats. In addition, species richness increased in proximity to tree groves (*p* < 0.05). Our data also revealed that smaller distance to anthropogenic areas (*p* = 0.06) and single trees (*p* = 0.06) tended to benefit species richness, while species richness tended to decrease with greater proximity to standing water (*p* = 0.06). Similarly, bat activity increased significantly with greater forest land cover (*p* < 0.001) and smaller distance to forested areas (*p* < 0.01). Bat activity tended to increase with smaller distance to anthropogenic areas (*p* = 0.06) but to decrease at grassland site closer to standing water (p = 0.06).

**Table 1:** Statistical results of the Poisson distributed generalized linear mixed effect models (GLMM). Presented are landscape elements and their respective effect on bat occurrence and bat activity on grassland recording sites in the Schorfheide-Chorin.

GLMM-Model	Landscape elements	Estimate	Error	Z value	*P* > (|Z|)
(a)					
*Species Richness*	*Intercept*	*1*.*9*	*1*.*1* ^*−01*^	*18*.*4*	***
Poisson	Forest area	7.1^−01^	2.2^−01^	3.2	**
AIC: 406.6	Anthropogenic area	1.1	9.2^−01^	2.3	*n*.*s*.
Deviance: 384.6	Distance to forest	-6.8^−04^	3.3^−04^	-2.1	*
	Distance to anthropogenic areas	-2.3^−04^	1.2^−04^	-1.9	.
	Distance to groves	-2.2^−04^	1.1^−04^	-2.0	*
	Distance to linear vegetation	-2.8^−04^	4.1^−04^	-0.7	*n*.*s*.
	Distance to single trees	-5.4^−04^	2.9^−04^	-1.9	.
	Distance to standing water	1.2^−04^	6.6^−05^	1.9	.
	Distance to running water	1.7^−04^	2.2^−04^	0.8	*n*.*s*.
(b)					
*Activity*	*Intercept*	*3*.*0*	*2*.*2* ^*−01*^	*13*.*4*	***
(Poisson)	Forest area	2.2	4.9^−01^	4.5	***
AIC: 3846	Anthropogenic area	2.0	2.1	1.0	*n*.*s*.
Deviance: 3824	Distance to forest	-1.8^−03^	6.7^−04^	-2.6	**
	Distance to anthropogenic areas	-4.7^−04^	2.5^−04^	-1.9	.
	Distance to groves	-1.1^−04^	2.3^−04^	-0.5	*n*.*s*.
	Distance to linear vegetation	-7.5^−04^	8.4^−04^	-0.9	*n*.*s*.
	Distance to single trees	-8.3^−04^	5.9^−04^	-1.4	*n*.*s*.
	Distance to standing water	2.8^−04^	1.5^−04^	1.9	.
	Distance to running water	8.8^−05^	4.7^−04^	0.2	*n*.*s*.

*n*.*s* = non significant

P < 0.1.;

*P* < 0.05 *

*P*<0.01 = **

*P*<0.001 = ***

**Fig 2:**
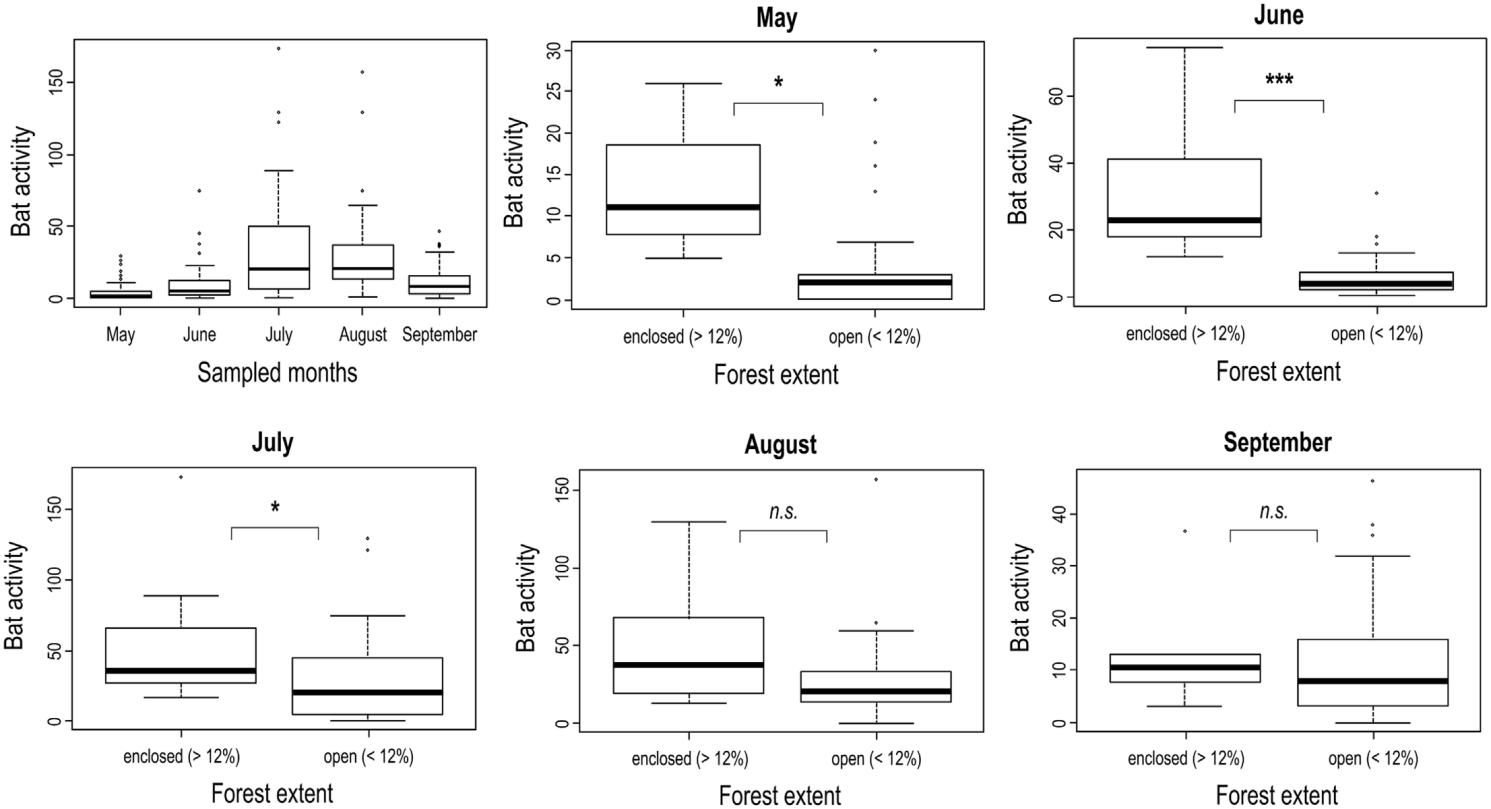
Bat activity on 50 grassland plots in relation to forest extent in the 200m buffer zone throughout five sampling months. n.s = non significant, P < 0.05 *, P < 0.001 = ***

Species richness and bat activity varied significantly throughout the five months of our study (Fig 2). Both were lowest in May and June, highest in July and August and decreased slightly in September. Moreover, the importance of forest area for bat activity varied over the months of data collection. Our data revealed a higher bat activity on grasslands with greater forest land cover in the 200 m buffer in May, June and July, compared to August and September (for more details refer to Table 2 and Fig 2).

**Table 2:** Analysis of deviance table, listing the overall results of the Poisson distributed generalized linear mixed effect models (GLMM) investigating the seasonal importance of forest areas near grassland recording sites for bat species richness and activity.

		Forest area	*P*	Forest distance	*P*
*Species*	Month	LR χ^2^_4_ = 133.3	***	LR χ^2^_4_ = 137.9	***
*Richness*	Forest area	LR χ^2^_1_ = 25.8	***	LR χ^2^_1_ = 9.36	**
	Forest area* Month	LR χ^2^_4_ = 16.8	**	LR χ^2^_4_ = 5.5	*n*.*s*.
*Activity*	Month	LR χ^2^_4_ = 1272.6	***	LR χ^2^_4_ = 1252.6	***
	Forest area	LR χ^2^_1_ = 34.8	***	LR χ^2^_1_ = 9.2	**
	Forest area* Month	LR χ^2^_4_ = 132.8	***	LR χ^2^_4_ = 76.5	***

*n*.*s* = non significant

P < 0.1.

*P* < 0.05 *

*P*<0.01 = **

*P*<0.001 = ***

### Importance of landscape elements for bat species composition

**Fig 3:**
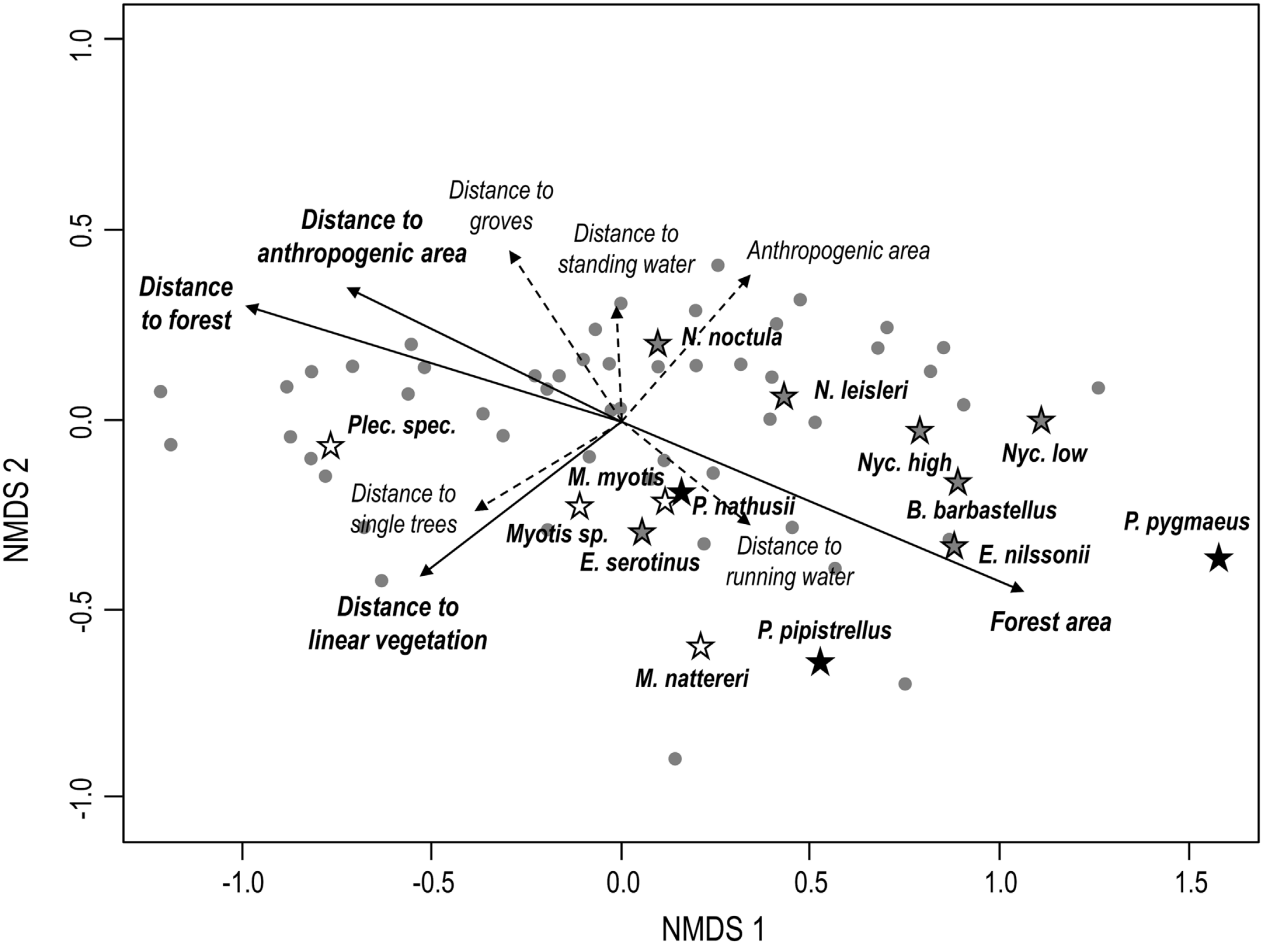
Ordination of the 50 different sampling plots in an NMDS space based on Bray-Curtis dissimilarity of bat species composition (weighted by the relative intensity of habitat use). Points represent the placement of plots and asterisks represent the placement of species within multidimensional space. The landscape variables explaining differences in species composition between plots are represented as vectors and were fitted using the function envfit (R-package vegan, [50]). Significantly important landscape variables have solid lines, the others have dashed lines.

Non-metric multidimensional scaling (NMDS, final stress = 0.08, linear fit *r* ^2^ = 0.97) clearly separated recording sites based on Bray-Curtis dissimilarities in species composition (Fig 3). Sampling plots with greater forest land cover in their vicinity were separated from grassland plots in less structured landscapes along the NMDS axis 1, indicating distinct differences in bat species composition along a gradient from forested areas to open landscapes. In addition, the distance to standing water and linear vegetation mainly separated sampling plots along the NMDS axis 2. In particular forest land cover (*r* ^2^ = 0.3865, *p* < 0.001), the distance to forests (*r* ^2^ = 0.3139, *p* < 0.01), the distance to anthropogenic areas (*r* ^2^ = 0.1903, *p* = 0.0140), and the distance to linear vegetation (*r* ^2^ = 0.1303, *p* < 0.05) significantly explained differences in bat species composition between grassland plots.

The distance to forest (Adonis, F = 10.65, p< 0.001), distance to grooves (Adonis, F = 7.47, p< 0.01) and distance to standing water (Adonis, F = 7.1, p< 0.01) also explained differences in the species richness of functional groups on grassland sites.

## Discussion

Land use and land use intensification are considered as the most dominant factors driving biodiversity loss [51–52]. Nevertheless, an enrichment of the landscape by landscape elements such as vegetation patches, corridors or single trees as stepping stones can offset the depletion of species and help maintain biodiversity and ecosystem function in production landscapes [53]. However, the importance of such landscape elements varies between species and functional groups due to different requirement for certain landscape features [25]. It is thus of high interest to land owners, managers and conservationists to understand which landscape elements are important and sufficient to assure high species richness and thus vital ecosystem function (e.g., pest control) in agricultural areas.

Insufficiently connected landscape elements might hinder species to capitalize on potential resources [24] within homogenous agricultural landscapes. Our results on bats, which are highly mobile animals, revealed that this is already true on a very small scale. All our recording sites were closer than 500 m in distance to forested areas and closer than 1500 m to villages or settlements which potentially provide roosting sites for bats. Nevertheless, our data revealed a significant increase in bat activity and species richness on grassland sites closer to forests and with greater forest land cover in the immediate surroundings. Despite the small distances to forest remnants our data also indicated a high value of tree groves for species richness of bats on grassland sites. This is in accordance with previous findings, where scattered trees in rural landscapes revealed a high importance for bat activity and species richness (e.g. [16, 22, 33, 35, 54]) and underlines the importance of groves and even single trees as stepping stones [53] for flying mammals such as bats. Thus, our results highlight that a dense network of connecting elements within the landscape is essential to assure the use of agricultural landscapes as foraging areas, even for highly mobile species such as bats.

Our results revealed that the importance of landscape features for bat activity in agricultural areas can vary over time. In particular, forest land cover, and closer proximity to forest areas revealed a positive influence on bat activity in spring. Potentially, nearby forest areas buffer microclimatic conditions, which is reflected in warmer minimum temperatures and cooler maximum temperatures in such grasslands [55]. Thus, grassland sites adjacent to forest remnants experience less seasonal variability in climatic conditions and thus might be especially valuable foraging sites for bats in the beginning of the year.

As expected, the landscape features in the direct proximity to our recording sites had a significant effect on bat species composition. Species-specific activity levels mainly differed between plots in closer vicinity to forest and anthropogenic areas from grassland plots isolated within open landscapes. While, for example, the open space foraging *Nyctalus noctula* revealed high activity levels in open space, slower flying species such as *Pipistrellus pipistrellus* were mostly active with greater forest land cover (Fig 3). However, in contrast to our expectations, our data revealed that species richness of all functional groups was highest at sites with larger amount of forest land cover in their direct surroundings and at closer proximity to forest patches. As species richness in general favors functional diversity, this very likely has direct implications for ecosystem resilience in agricultural landscapes [56]. Thus, agricultural areas closer to forested areas may be better buffered and likely more resilient against disturbances, such as outbreaks of potential pest insects [29] due to the increased presence and species richness of bats as predators of herbivorous insects.

## Supporting Information

S1 Dataset Landscape composition in the direct neighborhood of acoustic recording sites. Closest distance measures of landscape elements to acoustic recording sites and percent land cover of forest and urban settlements in a 200 m buffer around acoustic recoding stations.
(XLSX)

S2 Dataset Acoustic recording data of aerial insectivorous bats in the Schorfheide-Chorin. Bat activity (passes per hour), species richness and number of species within functional groups at each recording station during monthly surveys.
(XLSX)

S1 Fig Sonograms of typical search flight echolocation calls of bat species recorded above grassland sites in the Schorfheide-Chorin. Recorded echolocation calls were either qcf- (quasi-constant frequency) calls or combinations of downward modulated fm- (frequency modulated) and qcf- (quasi-constant frequency) call components. Echolocation calls of ten species (*Nyctalus noctula*, *N*. *leisleri*, *Eptesicus serotinus*, *E*. *nilssonii*, *Barbastella barbastellus*, *Pipistrellus nathusii*, *P*. *pipistrellus*, *P*. *pygmaeus*, *Myotis myotis* and *M*. *nattereri*) were identified with high certainty to species level. Echolocation calls with a high similarity in call structure were grouped into 4 sonotypes (*Nyctaloid low*, *Nyctaloid high*, *Plecotus spec*., and *Myotis spec*.). Please refer to the section ‘Species identification’ in the Materials and Methods part for further information.
(TIF)
