# Supplementary figures and images for: The Importance of Landscape Elements for Bat Activity and Species Richness in Agricultural Areas
Source: PLoS One. 2015 Jul 31;10(7):e0134443. doi: 10.1371/journal.pone.0134443 (PMC4521758; doi:10.1371/journal.pone.0134443)

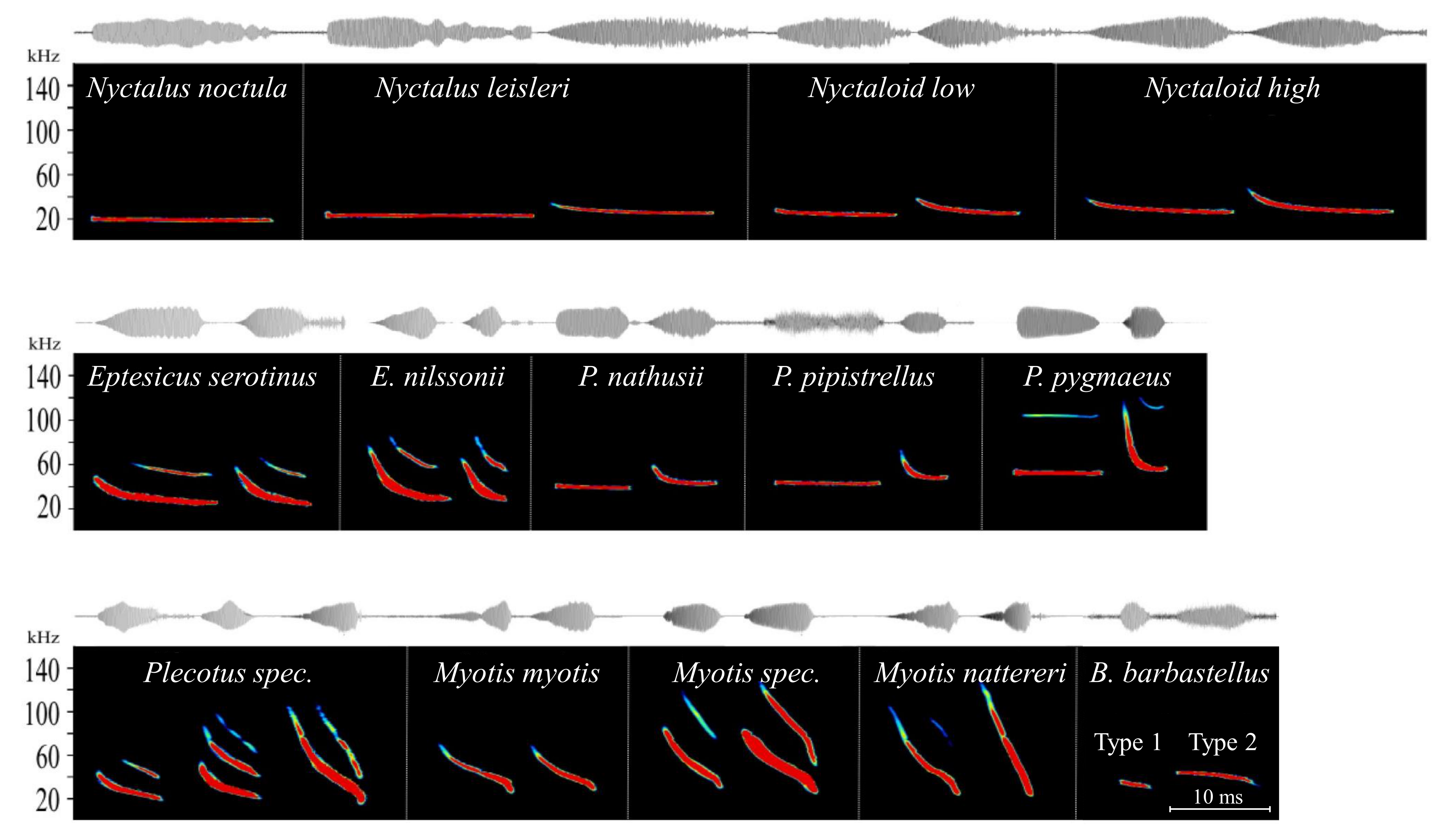

Supplement: S1 Fig — Recorded echolocation calls were either qcf- (quasi-constant frequency) calls or combinations of downward modulated fm- (frequency modulated) and qcf- (quasi-constant frequency) call components. Echolocation calls of ten species (Nyctalus noctula, N. leisleri, Eptesicus serotinus, E. nilssonii, Barbastella barbastellus, Pipistrellus nathusii, P. pipistrellus, P. pygmaeus, Myotis myotis and M. nattereri) were identified with high certainty to species level. Echolocation calls with a high similarity in call structure were grouped into 4 sonotypes (Nyctaloid low, Nyctaloid high, Plecotus spec., and Myotis spec.). Please refer to the section ‘Species identification’ in the Materials and Methods part for further information. (TIF) [file pone.0134443.s003.tif]
